# Editorial

**Published:** 1884-06

**Authors:** 


### EDITORIAL.

In many of our best general educational institutions of the country, provision is made for those who desire to pursue studies after having obtained a diploma, or diplomas; this usually is no reflection upon the course of instruction, nor the manner in which that instruction is given; but it is because more extended knowledge is desired than is embraced in the regular course. I am not aware that provision has been made in any professional schools for such advanced work, except that hospital work for medical students may be so regarded. Why provision has not been made for such work in professional schools it is not easy to see, for it will be admitted by almost every one, that those who go out from such school are as not well prepared for thorough work as they ought to be. Graduates of our dental colleges, many of them at least, realize this fact upon first assuming the work of their profession; and many of them express a desire for further opportunity for preparation.

In order that some opportunity may be afforded to its graduates the authorities of the "Dental College of the University of Michigan" have decided to arrange for such advanced study. Facilities will, therefore, be made for such work during the next regular term, and it is confidently expected that quite a number will enter upon it. Any information concerning this matter will be given by application to the Dean.

